# Sex‐moderated relationship between the 2D:4D ratio and circulating hormones in an adult Ghanaian population

**DOI:** 10.14814/phy2.15578

**Published:** 2023-01-25

**Authors:** Moses Banyeh, Samuel Kwasi Appiah, Abdul‐Malawi Abubakari, Musah Issah, Nafiu Amidu

**Affiliations:** ^1^ Department of Biomedical Laboratory Science University for Development Studies Tamale Ghana

**Keywords:** adult, digit ratios, estradiol, Ghana, testosterone

## Abstract

The second‐to‐fourth digit ratio (2D:4D) is the putative marker of prenatal hormone exposure. The 2D:4D ratio or the right–left difference (Dr‐l) are said to be negative and positive correlates, respectively, of circulating testosterone and estrogen in both adult males and females. However, previous studies on the subject have reported mixed results. This study aimed to determine the sex‐moderated relationship between the 2D:4D ratio and adult circulating testosterone, estradiol, testosterone‐to‐estradiol ratio and the free androgen index. This was a cross‐sectional study from January to June 2021 at the University for Development Studies, Ghana. The study involved 62 participants (Female = 28; Male = 34), aged between 20 and 26 years. The right (2D:4DR), the left (2D:4DL), and their difference (Dr‐l) were measured by computer‐assisted analysis. Fasting venous samples were assayed for total testosterone (T), estradiol (E_2_), and sex hormone‐binding globulin (SHBG) using ELISA. The free androgen index (FAI) was then calculated (T/SHBG) and the data were analyzed using moderated and/or weighted regression. Males had significantly higher T and FAI than females while females had significantly higher E_2_ than males, which were independent of age and body mass index (*p* < 0.001). There was a significant SEX*Dr‐l interaction on FAI (*p* = 0.007). The Dr‐l correlated negatively with FAI in males but positively in females and accounted for about 94.0% of the variability of FAI in males (adjR^2^ = 0.940) and only 0.2% in females (adjR^2^ = 0.002). The 2D:4D ratio, a putative marker of prenatal hormone exposure, may have an impact on sex differences in adult free androgen index.

## Graphical abstract

The relationship between the index of prenatal hormone exposure (2D:4D) and the free androgen index is moderated by sex.

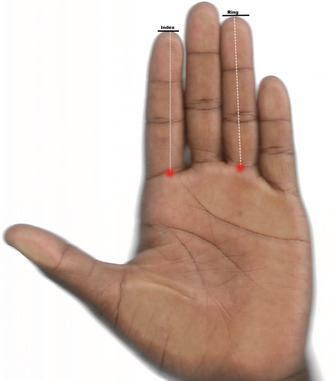

## 1 INTRODUCTION

The ratio of the second‐to‐fourth digit (2D:4D) is the putative marker of prenatal testosterone (PT) and estrogen (PE) exposure and has been suggested to be sexually dimorphic with low ratios in males than females on average (Manning, Scutt, et al., 1998). From amniotic fluid sample analysis, the 2D:4D ratio has been shown to be a negative correlate of PT, but a positive correlate of PE exposure in both males and females (Lutchmaya et al., 2004). Further evidence, from both humans and animals, of the relationship between the 2D:4D and PT exposure have demonstrated in conditions of altered steroidogenesis such as congenital adrenal hyperplasia, Klinefelter's syndrome, and androgen receptor gene polymorphisms (Manning, Bundred, et al., 2003; Richards et al., 2020; Zheng & Cohn, 2011). Asymmetry of the right–left 2D:4D ratio (Dr‐l) has also been demonstrated to be similar in pattern to the 2D:4D ratio. Studies have shown that low 2D:4D on the right hand is associated with more masculine traits while high 2D:4D on the left hand is related to more feminine traits such that a low Dr‐l is a marker of high PT and low PE exposure (Manning, 2002). This may be an indication that the right hand may be more responsive to PT and PE exposure than the left hand (Manning, 2002). It is, however, important to note that there is no consensus on findings as there are substantial overlaps between the sexes, smaller effect sizes in some studies, and no significant findings in the meta‐analysis (Hönekopp, 2013; Leslie, 2019; Nave et al., 2021). Notwithstanding, there are pieces of evidence in support of the 2D:4D ratio as the marker of prenatal androgenization in humans and an alternative to amniocentesis. A critical review and an overview of the literature has shown that the 2D:4D is a negative correlate of PT exposure (Mccormick & Carré, 2020; Swift‐Gallant et al., 2020).

It has been suggested that the 2D:4D ratio and/or the right–left difference (Dr‐l) are correlated with adult circulating testosterone and estrogen, an indication that PT and PE exposure may affect gonadal activity in adulthood (Manning, Scutt, et al., 1998; Manning, Wood, et al., 2004; Richards et al., 2018). If the relationship between 2D:4D and adult hormonal variables is true, then the validity of the 2D:4D ratio as a maker of prenatal androgenization will be problematic since the effect of PT and PE exposure on the 2D:4D ratio may be conflated by adult circulating hormones. However, previous studies, both cross‐sectional and longitudinal, have demonstrated that the 2D:4D ratio is established early in life and does not change afterward (Manning, 2002; Zheng & Cohn, 2011). The relationship between the 2D:4D and adult circulating hormones may stem from the fact that both originate from the same organs and can thus be correlated (Hönekopp et al., 2007; Trivers et al., 2006). Previous studies that examined the relationships between the 2D:4D ratio and/or their difference (Dr‐l) and adult circulating hormones have reported mixed outcomes. The 2D:4D and/or Dr‐l are a negative correlate of testosterone in males but a positive correlate of estrogen in females (Hönekopp et al., 2007; Manning, Wood, et al., 2004; Muller et al., 2011; Richards et al., 2018). However, some of the studies were characterized by methodological challenges which made their interpretation problematic. Some studies were conducted among clinical populations attending an infertility clinic while others were drawn from normative populations (Manning, Wood, et al., 2004, Richards et al., 2018). There was also, no homogeneity in the sample obtained for hormonal assays as some used serum/plasma while others used saliva or hair (Crewther et al., 2015; Richards et al., 2018). Moreover, some of the studies were conducted after the participants were subjected to a battery of challenging situations such as aggressive competitive sports while others did not. Yet, in one study, there was about a 10‐year gap between the time of blood draw and 2D:4D measurement (Crewther & Cook, 2019; Muller et al., 2011).

Research has shown that hormonal variables and the 2D:4D ratio show ethnic, population, and sub‐population variabilities (Manning et al., 2007; Manning, Fink, et al., 2022). Although the relationship between infant 2D:4D and maternal postpartum circulating hormones has been studied previously (Banyeh et al., 2021), there has not been any study in Ghana that has explored the relationship between the 2D:4D ratio and adult circulating hormones. This study aimed to determine the sex‐moderated relationship between the 2D:4D ratio and adult circulating testosterone, estradiol, testosterone‐to‐estradiol ratio, and the free androgen index.

## 2 MATERIALS AND METHODS

### 2.1 Study design and population

The study was cross‐sectional from January to June 2021 at the Tamale campus of the University for Development Studies (UDS). The university is multidisciplinary and multicampus and it is located in Tamale, the Northern Region of Ghana; it offers both undergraduate and postgraduate programs in Education, Biomedical, Medical, Nutritional Sciences, and Nursing/Midwifery. The study involved 62 participants (Female = 28; Male = 34), aged from 20 to 26 years. The participants were part of a larger study from which a part has been published (Banyeh et al., 2022). The participants were drawn from a normative population without a known history of fractures or conditions that could markedly affect digit lengths and standing height measurements. The participants had no known history of hormonal abnormalities or were not on hormonal therapy/contraceptives at the time of sampling. Participation was voluntary and was not restricted by a participant's religion, cultural group, or program of study.

### 2.2 Measurements

**FIGURE 1:**
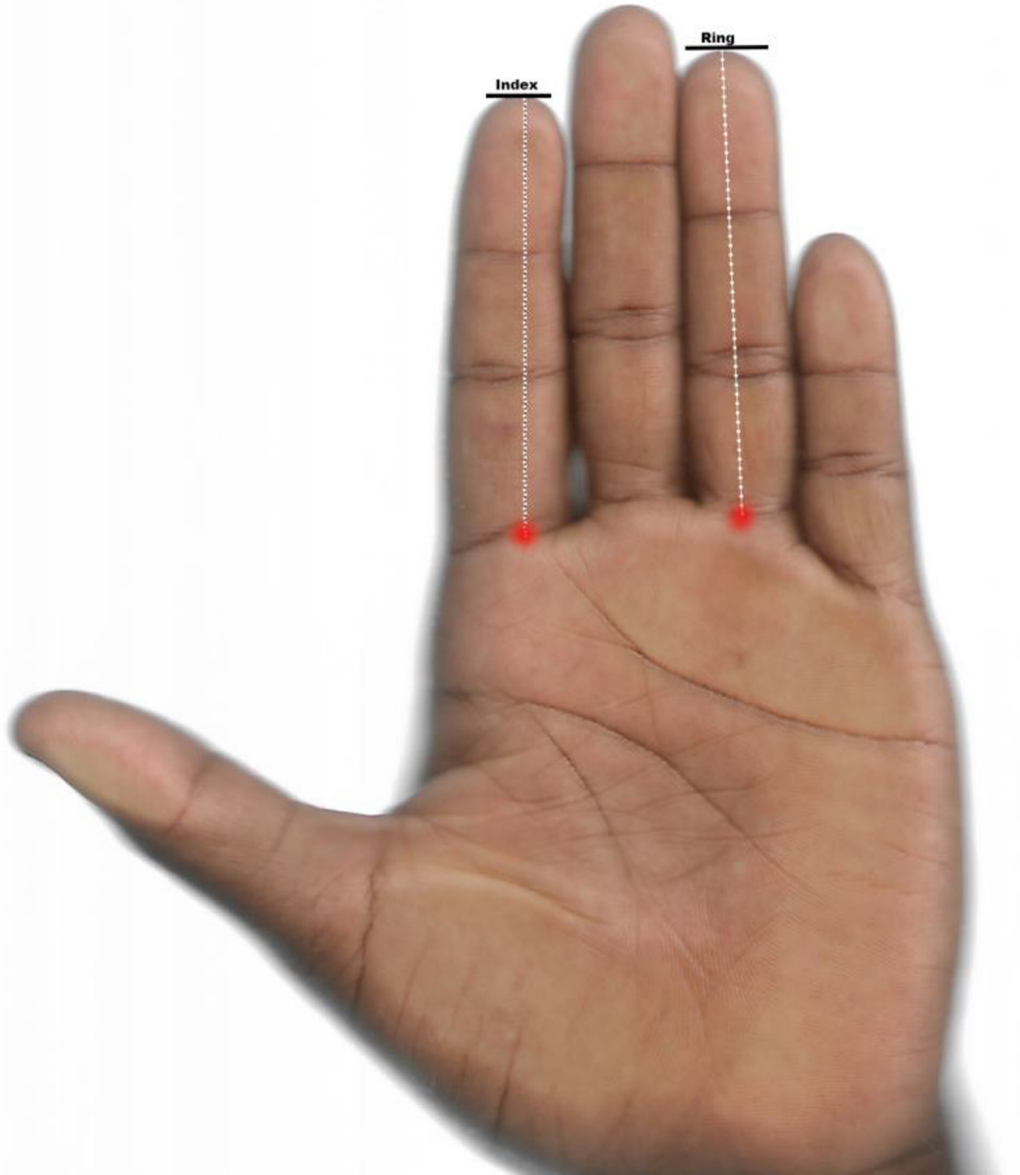
The scanned palmar surface of the hand shows the length of the second (index) and fourth (ring) fingers. The length of each finger was measured from the most proximal basal crease to the tip of the finger.

The standing height (cm) and body weight (kg) were measured following recommended guidelines using a stadiometer and bathroom scale, respectively (Gualdi‐Russo et al., 2018). The palmar surface of the right and left hands was scanned at 150 dpi according to anthropometric standards on the Hp desk jet 2620 all‐in‐one printer scanner (Allaway et al., 2009). The lengths of the second and fourth digits were measured using an electronic caliper in GIMP [(v 2.10.22), www.gimp.org] (Figure 1). Each measurement was taken twice by one observer and then averaged. The intraclass correlation coefficients (ICC) were calculated by employing the two‐way mixed, single measures with absolute agreement technique (Cuzzullin et al., 2022). The ICC were 0.950 and 0.966 for the 2D:4DR and 2D:4DL, respectively. The Dr‐l was derived from the differences between the right and the left 2D:4D ratios. A single venous blood sample was collected by venipuncture after an overnight fast (10–12 h) into a gel separator vacutainer tube. All blood samples were collected between 7:00 AM and 12:00 PM local time to avoid diurnal variations in circulating hormone levels (Hönekopp et al., 2007). The blood samples were allowed to clot for 30 min at 4°C before the tubes were then centrifuged at 1500 rpm for 10 min to separate the serum. The serum samples were aliquoted into 1.5 ml plastic cryotubes and then frozen at −25°C for later analysis. The samples were not previously thawed and refrozen. The serum total testosterone (T), estradiol (E_2_), and sex hormone‐binding globulin (SHBG) were analyzed in duplicates using the AccuBind® Microplate ELISA test system (Monobind Inc., Lake Forest, CA 92630, USA). The testing procedure followed the manufacturer's guidelines and the recommended reagents were used. The free androgen index (FAI) and the testosterone‐to‐estradiol ratio (T: E_2_) were derived by dividing T over SHBG and E2 (in the same unit), respectively.

### 2.3 Statistical analysis

The data were collected and analyzed in SPSS (v26) and GraphPad Prism (v8). The normality of the data and the presence of outliers were checked using the Shapiro–Wilk test. Descriptive statistics were then performed and parametric and nonparametric variables were summarized as mean ± SD and median (interquartile range—IQR), respectively. The sex differences in means and medians were determined using the student *t*‐test (two‐tailed) and the Mann–Whitney U test (two‐tailed) for parametric and nonparametric variables, respectively. The right–left 2D:4D ratio (Dr‐l) was compared to zero (0) using the one‐sample *t*‐test (one‐tailed) at a 90% confidence interval. The independent variables (2D:4D, Dr‐l) were first centered by subtracting the mean value from the variable to reduce multicollinearity (Robinson & Schumacker, 2009). Interaction variables were then created between the centered variable and the sex variable by multiplication (e.g., Sex*2D:4D‐centered). Linear regression models were then formulated with the hormonal variables as the dependent variables and the sex, 2D:4D‐centered, and the interaction variable (e.g., Sex*2D:4D) as predictors. Confounding was controlled by adding age and BMI as covariates into each model. The assumptions of multivariable linear regression were tested: Pearson correlation for a linear relationship between the dependent and the independent variables, variance inflation factor (VIF) for multicollinearity between the independent variables, Durbin–Watson test for autocorrelation and the Cook's distance (Cook's D) for influential multivariable outliers. The regression residuals were used to test the assumptions of multivariable normality and homoscedasticity using the frequency or probability–probability plots and scatter plots, respectively. Where heteroscedasticity was detected in a model with a significant main or interaction effect, a weighted regression was also performed (Yaffee, 2002). The unstandardized coefficient of the models with significant main or interaction effects was saved and used to plot graphs. The unstandardized predicted value of the dependent variable was plotted on the y‐axis, the centered predictor variable on the x‐axis, and sex was used as the marking variable. The statistical significance was set at *p* < 0.050.

## 3 RESULTS

### 3.1 Descriptive statistics

The descriptive statistics of the study population are shown in Table 1. The female participants had significantly higher circulating estradiol than males (*p* < 0.001). However, males had significantly higher testosterone, free androgen index (FAI), and testosterone‐to‐estrogen ratio (T: E2) than females (*p* < 0.001). There were no significant sex differences in the 2D:4D ratio or their difference.

**TABLE 1:** The general characteristics of the study population stratified by sex

Variables	Female *n* = 28	Male *n* = 34	*p*‐value
BMI (kg/m^2^)	20.7 ± 1.30	20.5 ± 1.47	0.547
T (nmol/ml)	1.46 (0.81–1.93)	21.18 (18.70–27.38)	<0.001
E_2_ (pg/ml)	61.07 (47.67–85.99)	34.41 (22.69–39.01)	<0.001
FAI	0.01 (0.005–0.015)	0.35 (0.141–0.736)	<0.001
T: E_2_	5.42 (3.82–9.38)	208.04 (150.03–341.61)	<0.001
2D:4DR	0.939 ± 0.043	0.934 ± 0.037	0.621
2D:4DL	0.940 ± 0.046	0.936 ± 0.039	0.729
Dr‐l	−0.001 ± 0.030	−0.002 ± 0.035	0.871

*Note*: The results are summarized as either mean ± SD or median (IQR) for parametric and nonparametric variables, respectively. Differences in means and medians were determined using the student's t‐test (unpaired, two‐tailed) and the Mann–Whitney U test (unpaired, two‐tailed), respectively. The Dr‐l was compared to zero (0) using the one‐sample t‐test (one‐tailed) at 90%CI.

Abbreviations: BMI, body mass index; CI, confidence interval; E_2_, estradiol; FAI, free androgen index; T, testosterone; T: E_2_, testosterone‐to‐estradiol ratio.

### 3.2 Univariable linear regression and correlation analysis

**FIGURE 2:**
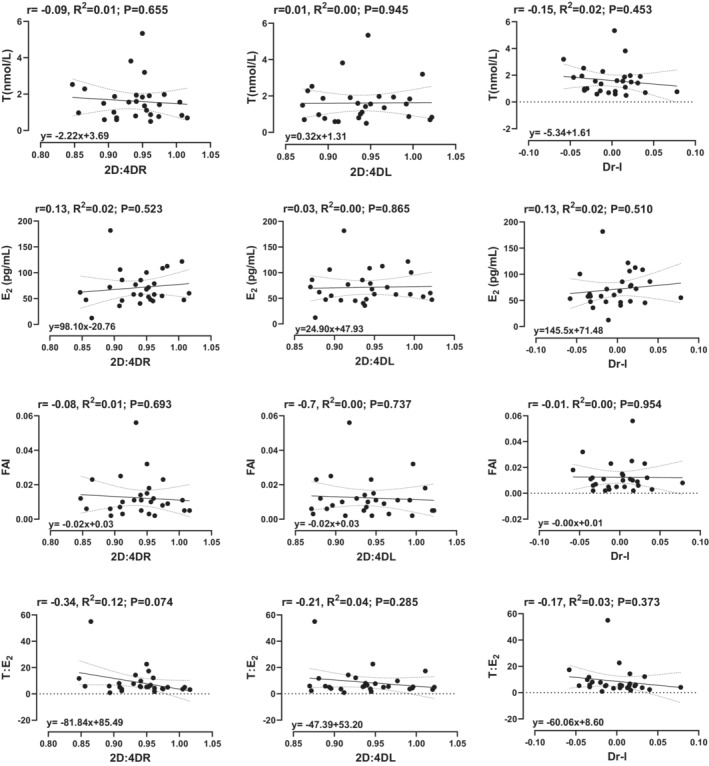
Univariable linear regression and correlation analysis between hormonal variables (dependent) and predictor variables (digit ratios) in females. T = total testosterone, E_2_ = estradiol, and FAI = free androgen index.

**FIGURE 3:**
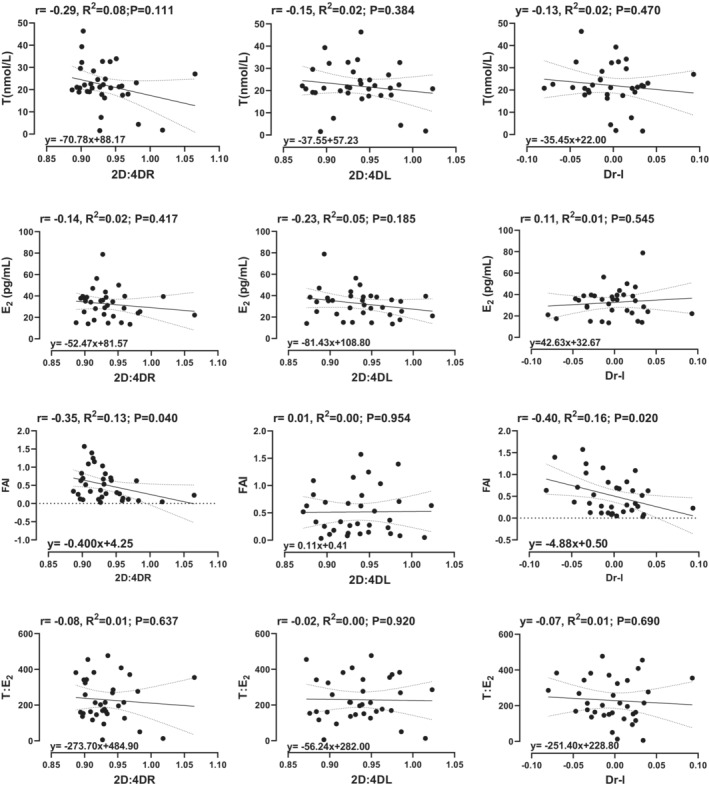
Univariable linear regression and correlation analysis between hormonal variables (dependent) and predictor variables (digit ratios) in males. T = total testosterone, E_2_ = estradiol, and FAI = free androgen index.

The univariable and correlational analysis between adult hormonal variables and the 2D:4D are shown in Figures 2 and 3. There was no significant correlation or relationship between adult female hormonal variables and the 2D:4D ratio (Figure 1). However, in adult males, the FAI was significantly correlated with the righthand 2D:4D ratio (*r* = −0.35, *p* = 0.040) and the Dr‐l (*r* = −0.40, *p* = 0.020). Moreover, for every unit increase in the FAI, there was a corresponding decrease of 0.400 in the righthand 2D:4D and 4.88 in the Dr‐l (Figure 2).

### 3.3 Sex‐2D:4D interactions on adult hormonal variables

**FIGURE 4:**
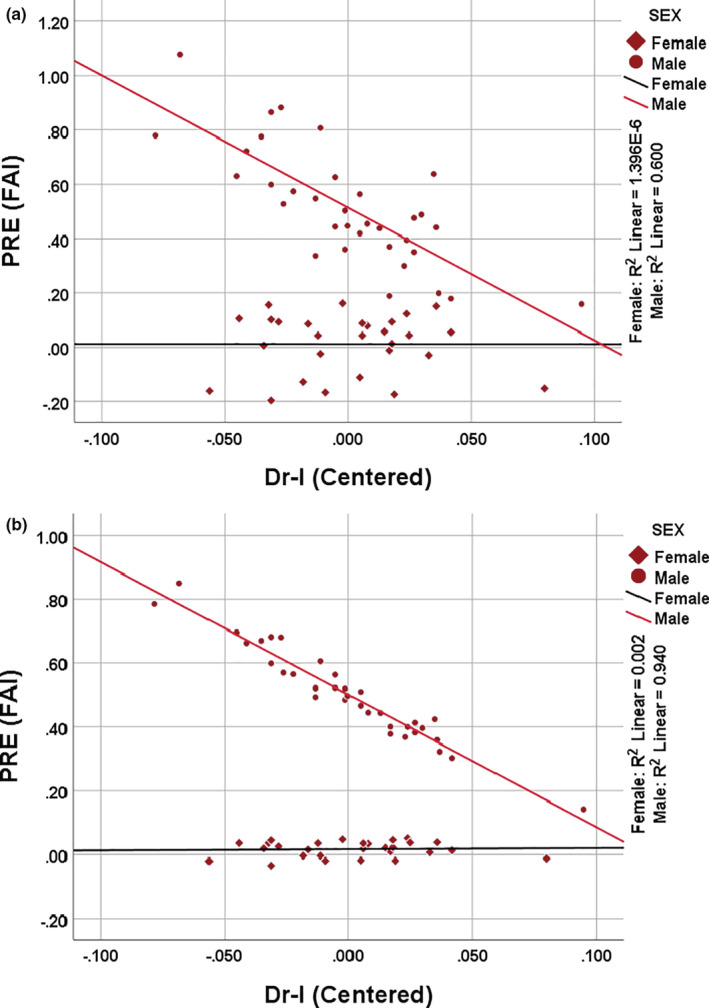
Moderated linear regression plot with interaction effects between sex and the Dr‐l on adult circulating free androgen index (FAI). The models were formulated using multivariable regression without a weight (a) and with a weight (b). PRE = unstandardized predicted value.

The sex‐moderated linear regression analyses between the 2D:4D ratio and hormonal variables are summarized in Tables 2, 3, 4 and Figure 4. There were significant sex differences in hormonal variables after adjusting for age and BMI. However, the 2D:4D ratio had no significant effect on adult circulating testosterone, estradiol, or their indices. But from LR‐11A of Table 4, there was a significant interaction between sex and the Dr‐l on the free androgen index (*p* = 0.047). Correcting for heteroscedasticity (LR‐11B of Table 4), there was a significant interaction between sex and the Dr‐l on FAI (*p* = 0.007). The FAI decreased with increasing Dr‐l which was independent of age and BMI. However, the relationship between FAI and Dr‐l was negative in males but positive in females. The Dr‐l accounted for about 94% (adjR^2^ = 0.940) and 0.2% (adjR^2^ = 0.002) of the variabilities in FAI in males and females, respectively, which were independent of age and BMI.

**TABLE 2:** Sex‐moderated regression models involving the righthand 2D:4D ratio

LR model	Variables	B	95% CI		*p*‐value	F‐value	AdjR^2^
			Lower	Upper			
1	**T (nmol/ml)**						
	(Constant)	−1.44	−44.80	41.93	0.947	26.932*	0.68
	AGE (years)	0.17	−1.07	1.41	0.789		
	BMI (kg/m^2^)	−0.03	−1.41	1.36	0.972		
	SEX	20.00	15.78	24.21	<0.001		
	2D:4DR	−2.48	−65.52	60.57	0.938		
	SEX*2D:4DR	−68.42	−160.20	23.36	0.141		
2	**E**_**2**_ **(pg/ml)**						
	(Constant)	33.08	−119.13	185.29	0.665	8.340*	0.38
	AGE (years)	−1.44	−5.79	2.91	0.510		
	BMI (kg/m^2^)	3.33	−1.53	8.18	0.175		
	SEX	−35.16	−49.96	−20.37	<0.001		
	2D:4DR	110.41	−110.87	331.69	0.322		
	SEX*2D:4DR	−125.77	−447.91	196.37	0.437		
3	**FAI**						
	(Constant)	−2.61	−4.34	−0.89	0.004	13.663*	0.51
	AGE (years)	0.06	0.01	0.11	0.028		
	BMI (kg/m^2^)	0.07	0.01	0.12	0.015		
	SEX	0.41	0.24	0.58	<0.001		
	2D:4DR	0.15	−2.37	2.66	0.908		
	SEX*2D:4DR	−3.28	−6.94	0.37	0.078		
4	**T: E**_**2**_						
	(Constant)	−544.39	−1100.01	11.23	0.055	19.291*	0.60
	AGE (years)	11.29	−4.59	27.16	0.160		
	BMI (kg/m^2^)	15.05	−2.66	32.76	0.094		
	SEX	203.22	149.21	257.24	<0.001		
	2D:4DR	−44.94	−852.69	762.82	0.912		
	SEX*2D:4DR	−41.89	−1217.81	1134.04	0.943		

*Note*: The 2D:4DR was centered on its mean before the interaction variable was formulated. The models were adjusted for age and BMI.

Abbreviations: BMI, body mass index; CI, confidence interval; E_2_, estradiol; FAI, free androgen index; T, testosterone; T:E_2_, testosterone‐to‐estradiol ratio.

* Significant at *p* < 0.001.

**TABLE 3:** Sex‐moderated regression models involving the lefthand 2D:4D ratio

LR model	Variables	B	95%CI		*p*‐value	F‐value	AdjR^2^
			Lower	Upper			
5	**T (nmol/ml)**						
	(Constant)	−7.11	−51.30	37.07	0.748	24.900*	0.66
	AGE (years)	0.22	−1.05	1.49	0.729		
	BMI (kg/m^2^)	0.19	−1.21	1.60	0.784		
	SEX	20.05	15.72	24.38	<0.001		
	2D:4DL	0.82	−60.66	62.31	0.979		
	SEX*2D:4DL	−36.56	−125.96	52.83	0.416		
6	**E**_**2**_ **(pg/ml)**						
	(Constant)	38.68	−113.03	190.39	0.612	8.130*	0.37
	AGE (years)	−1.48	−5.85	2.89	0.500		
	BMI (kg/m^2^)	3.11	−1.71	7.93	0.202		
	SEX	−35.45	−50.32	−20.59	<0.001		
	2D:4DL	39.45	−171.67	250.58	0.710		
	SEX*2D:4DL	−96.95	−403.90	210.01	0.530		
7	**FAI**						
	(Constant)	−3.12	−4.91	−1.34	0.001	11.660*	0.47
	AGE (years)	0.06	0.01	0.11	0.021		
	BMI (kg/m^2^)	0.09	0.03	0.15	0.003		
	SEX	0.42	0.24	0.59	<0.001		
	2D:4DL	0.27	−2.22	2.75	0.832		
	SEX*2D:4DL	0.64	−2.98	4.25	0.726		
8	**T: E**_**2**_						
	(Constant)	−566.05	−1116.90	−15.21	0.044	19.287*	0.60
	AGE (years)	11.50	−4.36	27.36	0.152		
	BMI (kg/m^2^)	15.87	−1.62	33.37	0.074		
	SEX	203.51	149.53	257.49	<0.001		
	2D:4DL	2.40	−764.18	768.97	0.995		
	SEX*2D:4DL	83.72	−1030.81	1198.25	0.881		

*Note*: The 2D:4DL was centered on its mean before the interaction variable was formulated. The models were adjusted for age and BMI.

Abbreviations: BMI, body mass index; CI, confidence interval; E_2_, estradiol; FAI, free androgen index; T, testosterone; T: E_2_, testosterone‐to‐estradiol ratio.

* Significant at *p* < 0.001.

**TABLE 4:** Sex‐moderated regression models involving the Dr‐l variable

LR model	Variables	B	95%CI		*p*‐value	F‐value	AdjR^2^
			Lower	Upper			
9	**T (nmol/ml)**						
	(Constant)	−9.90	−53.65	33.85	0.652	24.719*	0.66
	AGE (years)	0.26	−1.02	1.53	0.689		
	BMI (kg/m^2^)	0.29	−1.09	1.67	0.674		
	SEX	20.05	15.71	24.39	<0.001		
	Dr‐l	−5.92	−99.19	87.35	0.899		
	SEX*Dr‐l	−28.63	−147.64	90.39	0.632		
10	**E**_**2**_ **(pg/ml)**						
	(Constant)	34.18	−115.00	183.36	0.648	8.303*	0.37
	AGE (years)	−1.39	−5.73	2.95	0.523		
	BMI (Kg/m2)	3.23	−1.48	7.95	0.175		
	SEX	−35.41	−50.20	−20.62	<0.001		
	Dr‐l	135.79	−182.24	453.81	0.396		
	SEX*Dr‐l	−82.70	−488.53	323.12	0.685		
11A	**FAI**						
	(Constant)	−2.90	−4.51	−1.28	0.001	16.171*	0.55
	AGE (years)	0.06	0.01	0.11	0.015		
	BMI (kg/m^2^)	0.08	0.03	0.13	0.003		
	SEX	0.41	0.25	0.57	<0.001		
	Dr‐l	−0.17	−3.61	3.27	0.921		
	SEX*Dr‐l	−4.46	−8.84	−0.07	0.047		
11B	**FAI**						
	(Constant)	−0.69	−1.57	0.19	0.124	18.763*	0.59
	AGE	0.02	−0.01	0.05	0.210		
	BMI	0.01	−0.01	0.04	0.259		
	SEX	0.45	0.34	0.57	<0.001		
	Dr‐l	0.02	−1.05	1.08	0.975		
	SEX*Dr‐l	−4.13	−7.10	−1.15	0.007		
12	**T: E**_**2**_						
	(Constant)	−552.42	−1095.63	−9.21	0.046	19.385*	0.60
	AGE (years)	11.36	−4.45	27.16	0.156		
	BMI (kg/m^2^)	15.36	−1.80	32.53	0.078		
	SEX	203.29	149.43	257.16	<0.001		
	Dr‐l	−92.20	−1250.23	1065.83	0.874		
	SEX*Dr‐l	−111.30	−1589.03	1366.44	0.881		

*Note*: The Dr‐l was centered on its mean before the interaction variable was formulated. The models were adjusted for age and BMI.

Abbreviations: A, moderated linear regression; B, moderated linear regression with weight; BMI, body mass index; CI, confidence interval; E_2_, estradiol; FAI, free androgen index; T, testosterone; T: E_2_, testosterone‐to‐estradiol ratio.

* Significant at *p* < 0.001.

## 4 DISCUSSION

This study sought to determine sexual dimorphism and sex‐2D:4D interactions on the levels of adult circulating testosterone and estradiol. There were substantial sex differences in circulating hormonal variables which were independent of age and BMI. Males had markedly higher T, FAI, and T: E2 than females and vice‐versa regarding E_2_. Also, no sex differences in the 2D:4D ratio or their difference (Dr‐l) were observed. In males, there was an inverse relationship between the FAI and that of the 2D:4DR and the Dr‐l. Moreover, there was a noticeable interaction between sex and the Dr‐l on FAI such that males' FAI decreased with increasing Dr‐l while that of females slightly increased with increasing FAI.

No significant relationship between 2D:4D and the total T or E_2_ was observed. This was consistent with previous studies, including meta‐analysis, which found no strong or significant association between the 2D:4D ratio and adult circulating hormones (Beaton et al., 2011; Folland et al., 2012; Hönekopp et al., 2007; Muller et al., 2011; Ronay et al., 2018; Zhang et al., 2020). However, a previous study had observed that the right 2D:4D of adult males was negatively correlated with circulating testosterone while the 2D:4D ratio of both hands of females positively correlated with circulating estrogen levels which were independent of body weight and standing height (Manning, Scutt, et al., 1998). Similarly, significant relationships between the 2D:4D and adult circulating T or E_2_ had also been reported by other authors (Borráz‐León et al., 2018; Crewther et al., 2015; Klimek et al., 2014; Malik & Malik, 2011). It is, however, important to note that there are variabilities in methodology and sample population between studies which could have accounted for differences in their findings. Some previous studies were conducted among clinical samples while others were drawn from a healthy or normative population (Folland et al., 2012; Manning, Wood, et al., 2004; Manning, Scutt, et al., 1998). Also, there were variabilities in the samples used for the hormone measurements, ranging from blood, hair, and saliva (Crewther & Cook, 2019; Klimek et al., 2014; Manning, Scutt, et al., 1998; Manning, Wood, et al., 2004; Richards et al., 2018, Ronay et al., 2018). Some studies obtained samples after subjecting the participants to a battery of exercises, activities, or challenging situations (Crewther & Cook, 2019; Ribeiro et al., 2016). In one study, involving women, the time lapse between the drawing of blood samples for hormonal tests and the measurement of the 2D:4D ratio was over 10 years. In the same study, the blood samples were drawn at premenopause while the digit ratios were measured at postmenopause.(Muller et al., 2011).

Although there were no significant relationships between the 2D:4D and the Dr‐l with the total T and E_2_, the Dr‐l showed a negative relationship with the FAI in males but positively in females. This means that men whose Dr‐l was more negative (right < left 2D:4D) had higher FAI than those whose Dr‐l was more positive (right > left 2D:4D). The reverse was however observed in females. The FAI is the ratio of the total circulating T and the sex hormone‐binding globulin in the same unit. Manning, Stewart, et al. (2004) observed that men with lower 2D:4D on the right than the left had higher testosterone levels and vice‐versa. However, this observation was only made among men whose testicular function was likely compromised but was not observed among men drawn from a normative sample. Contrary to this study, a previous study found that asymmetry in digit ratio was found to be positively correlated with the circulating estradiol at each phase of the menstrual cycle and also the average estradiol in premenopausal women after controlling for multiple covariates. But salivary samples were used for the hormonal assay and not plasma or serum as was used in this study (Richards et al., 2018).

The Dr‐l, just like the 2D:4D, has been indicated to be a negative correlate of prenatal testosterone but a positive correlate of prenatal estrogen exposure, independent of the nation, sex, ethnicity, and age (Manning et al., 2007). The relationship between the Dr‐l and the structure of the androgen receptor gene is also suggestive of its association with testosterone (Manning, Wood, et al., 2004). Significant relation between the Dr‐l and adult testosterone or its calculated indices is indicative of the link between adult testicular cells and fetal Leydig cell activity, at least in infertile men (Manning, 2002; Manning, Scutt, et al., 1998). The Dr‐l may also be associated with changes or spikes in testosterone levels following challenging situations such as in aggressive contact sports. This may suggest that prenatal hormone exposure may prime the adult endocrine system to respond to challenging situations with the release of testosterone (Crewther et al., 2015; Crewther & Cook, 2019). However, it has been problematic to replicate studies suggestive of the relationship between Dr‐l and testosterone in challenging situations which some authors think is due to methodological challenges in accurately measuring the Dr‐l. The error inherent in 2D:4D arises from errors in 2D and 4D, but in Dr‐l, the errors arise from the 2D and 4D of both the left and right hands, requiring repeated measurements to achieve a good margin of accuracy (Ribeiro et al., 2016).

This study has some strengths. This study is the first in Ghana to examine possible interactions between sex and the 2D:4D ratio on adult circulating hormonal variables. Also, the assumptions of multivariable linear were tested for models of interest (Supplementary material). Assumption testing is recommended to determine model fitness and reliability (Robinson & Schumacker, 2009). Moreover, the digit ratios were measured by computer‐assisted analysis which is a more precise technique than direct measurements or using photocopies (Fink & Manning, 2018). The authors, however, acknowledge that the study numbers are relatively low which has implications for the interpretation of the results. Also, there are population variabilities in 2D:4D and hormonal variables due to genetic and environmental factors which may not allow for the generalization of results. Further population and subpopulation studies, with larger study numbers, are therefore recommended.

## 5 CONCLUSION

This study showed that the 2D:4D ratio was not significantly correlated with the total adult circulating testosterone or estradiol. However, the right–left difference (Dr‐l) showed a significant interaction with sex on adult‐free androgen index such that the relationship between the Dr‐l and FAI was negative in males but positive in females. Prenatal hormone exposure, as indexed by the Dr‐l, may have an impact on sex differences in adults' free androgen index.

## FUNDING INFORMATION

No funds, grants, or other support was received.

## CONFLICT OF INTEREST

The authors declare that there is no competing interest.

## AUTHORS' CONTRIBUTIONS

**Moses Banyeh and Nafiu Amidu**: conceptualization, methodology, supervision, interpretation of data, and project administration. **Abdul‐Malawi Abubakari, Samuel Kwasi Appiah, and Musah Issah:** investigation and data acquisition. **Moses Banyeh**: formal analysis and writing—original draft. All authors: writing—review, editing, and final approval before submission.

## ETHICAL DECLARATION

All the study procedures conformed with the guidelines of the Declaration of Helsinki (1964) and its subsequent amendments regarding human subject studies. The study was approved by the institutional review board of the University for Development Studies. Written informed consent was obtained from each participant before the study.

## Supporting information


Supplementary Material

## Data Availability

The data supporting this study will be available through the corresponding author upon reasonable request.
