# Supplementary material for: Sex‐moderated relationship between the 2D:4D ratio and circulating hormones in an adult Ghanaian population
Source: Physiol Rep. 2023 Jan 25;11(2):e15578. doi: 10.14814/phy2.15578 (PMC9875815; doi:10.14814/phy2.15578)
Supplement: Supplementary file 1 — Supplementary Material [file PHY2-11-e15578-s001.docx]

**Supplementary material**

**Sex-moderated relationship between the 2D:4D ratio and circulating hormones in an adult Ghanaian population**

**Assumptions of multivariable linear regression**

The assumptions of multivariable linear regression were tested as recommended, to check the fitness of model (Alita et al., 2021) (Figures 2 and 3 with their regression residuals: Supplementary Figures S1 and S2). The assumptions of multivariable linear regression for the unweighted (LR-11A) and weighted (LR-11B) regression models between FAI and Dr-l were tested. The Durbin-Watson tests value were 1.58 and 1.53 respectively for LR-11A and LR-11B. The variance inflation factor ranged from 1.06-2.62 and 1.02-1.19 respectively for LR-11A and LR-11B. The Cook’s D for LR-11A and LR-11B were in the range of 0.00-0.12 and 0.00-0.08 respectively. There was multivariable normality in LR-11A, however, the assumption of homoscedasticity was violated (Supplementary Figure S3). Therefore, weighted regression was also performed (LR-11B). The assumption that there was no autocorrelation or multicollinearity between the predictor variables was met as the Durbin-Watson tests were between 1.50 and 2.50 while the variance inflation factors were below ten (10) (Tranmer and Elliot, 2008). Also, the assumption that there are no influential multivariable outliers was met as the Cook’s D were less than 1.0 or 4/N (N=number of variables in the model i.e. 4/5=0.80)(Tranmer and Elliot, 2008). Moreover, the frequency distribution of the regression residuals and the probability-probability plot showed that there was multivariable normality (Noel et al.). The assumption of homoscedasticity was however violated as the distribution of regression residuals was not even about the zero mean in the scatter plot (Tranmer and Elliot, 2008). To overcome heteroscedasticity, a weight was calculated from the model residuals using auxiliary regression analysis. Although in weighted regression analysis in SPSS, residuals are not generated, it was observed that there was an improvement in the weighted regression model over the previous model when the Durbin-Watson, VIF and Cook’s D were compared as there was a marginal reduction in those values in the latter model.

**Supplementary Figure S1.** The regression residuals of the relationship between adult female hormonal variables and their digit ratios. T=total testosterone, E_2_=estradiol, FAI=free androgen index.

**Supplementary Figure S2.** The regression residuals of the relationship between adult male hormonal variables and their digit ratios. T=total testosterone, E_2_=estradiol, FAI=free androgen index.

**Supplementary Figure S3.**  The assumption of multivariable normality was tested using both the frequency (top) and the probability-probability (middle) plot. Homoscedasticity was tested using the scatter plot (bottom)
